# The impact of nurse managers’ boundary spacing leadership on the relationship between nurses’ work embeddedness and innovative work behaviors

**DOI:** 10.1186/s12912-024-02402-0

**Published:** 2024-10-25

**Authors:** Marwa Samir Sorour, Sally Mohammed Farghaly Abdelaliem, Sabrein Ali Khalifa Khattab

**Affiliations:** 1 Lecturer of Nursing Administration, Faculty of Nursing, Tanta University, Tanta, Egypt; 2 Assistant Professor of Nursing Administration, Nursing Administration Department, Faculty of Nursing, Alexandria University, Alexandria, Egypt

**Keywords:** Boundary Spacing Leadership, Nurses, Work Embeddedness, Innovative Work Behavior

## Abstract

**Aim:**

This study was designed to examine the nurse managers’ boundary-spacing leadership’s impact on the relationship between nurses’ work embeddedness and innovative work behaviors.

**Background:**

In today's evolving healthcare environment, innovation is essential for enhancing patient care, optimizing resources, and supporting healthcare professionals. Nurses are pivotal in driving bedside innovation, but cultivating a culture of innovation within nursing teams involves more than just promoting creative ideas.

**Methods:**

This is a descriptive correlational study that was conducted at one governmental hospital in Tanta, Egypt. Using Nurse Managers Boundary-Spacing Scale for assessing boundary-spacing leadership, The Global Job Embeddedness Scale for measuring work embeddedness, and Innovative Work Behavior Scale for assessing innovative work behavior, 250 nurses were surveyed. Data analysis was run using descriptive and inferential analysis. Correlation and regression analysis were used to examine the study hypotheses.

**Results:**

There is a statistically significant positive relationship between boundary-spacing leadership and work embeddedness (r = 0.615, *p* < 0.001). Boundary-spacing leadership accounted for 56% of the variance in work embeddedness and 36% of the variance in nurses' innovative work performance. These results highlight the pivotal role of boundary-spacing leadership in both enhancing nurses' work embeddedness and boosting their innovative behaviors. The substantial variance explained by boundary-spacing leadership underscores its critical influence on fostering a supportive and innovative work environment within the nursing field.

**Conclusion:**

Boundary spacing leadership style involves connecting internal and external resources, fostering information exchange, and advocating for the nursing team. While work embeddedness positively correlated with innovative work performance, its impact was less significant than boundary-spacing leadership. This suggests that feeling attached to the organization fosters innovation, but leadership style has a stronger influence. Furthermore, the study found a positive and significant correlation between innovative work performance and both work embeddedness and nurse managers' boundary spacing leadership.

**Practical Implications:**

The findings emphasize that boundary-spacing leadership is crucial for enhancing both nurses' work embeddedness and innovative behaviors. Healthcare organizations should focus on developing these leadership skills to create a supportive work environment, foster innovation, and improve staff retention

## Introduction

The healthcare sector is undeniably crucial due to its essential role in maintaining public health and well-being [1]. Within this sector, the concept of "boundary spacing" leadership holds significant weight for fostering innovation in nursing practices. This leadership style emphasizes the importance of relationship management for nurses and hospital staff [2]. Boundary spacing leaders act as filters, interpreting external situations and advocating for the hospital's needs, while simultaneously influencing external entities to prioritize the hospital's goals [2].

Nurse managers, in particular, play a vital role in enacting effective boundary-spacing leadership. Their efforts focus on building connections and overseeing interactions between their units, other departments within the hospital, and external stakeholders like patients, professionals, and external organizations [3]. The success of nursing practice often hinges on accessing resources and expertise from outside the immediate unit [4]. Head nurses who exhibit strong boundary-spacing leadership can facilitate nurses' access to opportunities, data, necessary support, and essential resources, positively impacting nurses' ability to connect and collaborate effectively [5]. This leadership style manifests through diverse activities, categorized into six key domains: informing and persuading, buffering, clarifying positions, connecting, feedback utilization, and cooperation and coordination [6]. Informing and persuading involves raising awareness about a unit's goals, activities, and values among external stakeholders. Buffering protects nurses from excessive external pressures and demands, shielding them from stress and overburdening [7]. These are just a few examples of how boundary spacing leadership, enacted by head nurses, can foster a supportive environment that promotes innovation within nursing teams.

The concept of boundary spacing leadership extends beyond informing and buffering. It encompasses crucial activities that promote collaboration, feedback utilization, and work embeddedness among nurses.Clarifying Positions involves clear communication of hospital and nursing department policies, decisions, assessments, and expectations to nurses. This enhances understanding and streamlines workflow within the unit [8]. Furthermore, head nurses who actively connect with external stakeholders can secure valuable support and resources for their unit, while encouraging nurses to access these resources effectively [8]. This highlights the leader's role in facilitating resource acquisition and knowledge sharing.

Feedback Utilization is another facet of boundary spacing leadership. Head nurses are critical in acquiring feedback from various professionals regarding the unit's performance [9]. This feedback can be instrumental in identifying areas for improvement and implementing changes that enhance patient care and overall unit function. Finally, Cooperation and Coordination of Activities is essential for a well-functioning healthcare environment. Head nurses who exhibit strong boundary spacing leadership actively seek collaboration and coordinate efforts with different units and professionals [10]. This fosters a more integrated approach to patient care, leverages expertise across departments, and ultimately improves the nursing unit's overall performance and work embeddedness [9, 10].

The concept of work embeddedness deserves further exploration in this context. Hospitals are complex organizations with the vital mission of delivering quality care to patients in critical situations [11]. Nurses who perceive a lack of organizational support, often stemming from ineffective communication or resource limitations, may experience negative feelings of work embeddedness towards their supervisors and the organization as a whole [12]. Work embeddedness, a relatively new concept, measures the degree of an individual's social integration within their organization [13]. It can also be understood as a "retention or anti-withdrawal construct" [14]. Rather than solely focusing on positive job attitudes, work embeddedness offers a broader perspective on why nurses choose to remain employed within a particular hospital. It encompasses the various forces influencing their decision to stay, including social connections, resource access, and a sense of value within the organization [15].

Work embeddedness, a concept gaining traction in healthcare research, offers valuable insights into nurse retention and innovative work behavior. This multi-dimensional construct encompasses three key aspects: nurse fit, nurse links, and nurse sacrifice [16]. Nurse fit refers to a nurse's perception of how well they align with their work environment and surrounding community [17]. This can encompass factors like compatibility with colleagues, patient population, and overall unit culture. Similarly, hospital work (organizational) fit focuses on a nurse's perception of how well they align with the hospital as a whole. A higher degree of perceived fit in both domains contributes to a stronger sense of work embeddedness [18].

Nurse links represent nurses' formal and informal connections within the hospital [19]. These connections can include relationships with colleagues, supervisors, other departments, and even patients. The strength and breadth of these links contribute to a sense of belonging and support within the organization. Nurse community sacrifice reflects the perceived ease with which these linkages can be disrupted [20]. Nurses who believe their connections are easily broken may be more likely to experience negative work embeddedness.

Nurse managers and head nurses play a crucial role in promoting work embeddedness among their teams [21]. Their efforts can focus on creating a healthy work environment, implementing strategies to improve nurse retention, and ultimately enhancing work performance. When nurses feel embedded within their organization, they are more likely to be engaged and motivated, contributing to a more positive work environment. Innovative work performance is a key outcome associated with a supportive work environment and strong work embeddedness [22]. It encompasses creating, introducing, and applying new ideas that benefit overall performance [22]. Creativity becomes a vital mechanism for nurses to thrive within the dynamic healthcare sector [23]. Nurse managers who foster a supportive environment can boost nurses' creativity by encouraging critical thinking and welcoming new ideas [21].

Innovative work performance can be further broken down into two key components: idea generation and idea realization [24]. Idea generation refers to finding and developing fresh solutions to problems, new workflows, and novel approaches to patient care [25]. This process relies heavily on individual traits like creativity, confidence, expertise, and a recognition of the need for improvement within the work environment. Idea realization, on the other hand, focuses on implementing these new ideas into practice, transforming them into new products, processes, or protocols within the hospital [22, 24]. This realization stage often involves collaboration with colleagues and can be influenced by factors beyond the individual nurse.

**Fig. 1:**
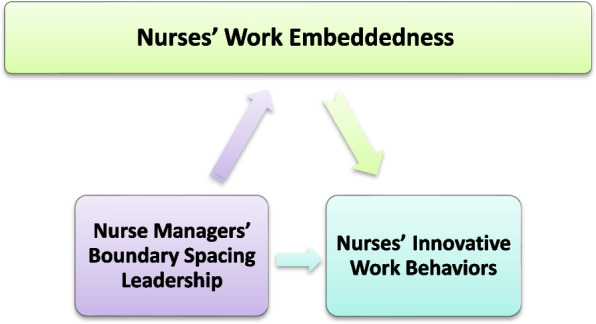
Conceptual Framework of the Study Variables

By understanding the relationship between work embeddedness, nurse leadership, and innovative work performance, healthcare organizations can implement strategies to create a more supportive work environment, promote nurse retention, and ultimately drive innovation within their teams (Fig. 1).

The model presented in this study aims to explore the influence of nurse managers' boundary-spacing leadership on the relationship between nurses' work embeddedness and their innovative work behaviors. The research seeks to determine how effective leadership that crosses organizational boundaries can enhance or modify the connection between how embedded nurses feel in their work environment and their ability to engage in innovative practices. By examining this relationship, the study aims to uncover whether boundary-spacing leadership by nurse managers can strengthen nurses' commitment to their roles (work embeddedness) while simultaneously promoting a culture of innovation, thereby improving overall healthcare outcomes.

## Synthesis of the research: current knowledge, gaps, and study contributions

### What is currently known:

Nurse managers play a crucial role in implementing boundary-spacing leadership, a style that involves connecting internal and external resources, facilitating information exchange, and advocating for the nursing team [26]. This leadership approach is essential for creating a supportive environment that encourages innovation among nurses. In parallel, work embeddedness, which refers to the extent of a nurse's integration within their organization, including factors such as fit, links, and sacrifices, is recognized as a significant contributor to nurse retention and job satisfaction [27]. Moreover, innovative work behaviors, encompassing both idea generation and realization, are vital for driving advancements in patient care and enhancing organizational performance. These behaviors are shaped by individual traits as well as the broader work environment [28]. Evidence suggests a positive interconnection between boundary-spacing leadership, work embeddedness, and innovative work behaviors. Specifically, boundary-spacing leadership has the potential to strengthen work embeddedness, which, in turn, fosters innovative behaviors among nurses, thereby highlighting the interconnectedness of these elements in promoting a culture of innovation in healthcare settings [29].

## What is unknown:

While boundary-spacing leadership is recognized for its importance in healthcare, the mechanisms through which it affects the relationship between work embeddedness and innovative work behaviors are not fully understood [30]. The direct and indirect impacts of this leadership style on innovation within nursing teams require further study. Although work embeddedness is linked to innovative behaviors, its relative importance compared to other factors like leadership style is unclear [31]. The nuanced ways in which work embeddedness contributes to innovation, especially in the context of boundary-spacing leadership, have not been thoroughly examined [32]. Additionally, the influence of contextual factors—such as organizational culture, resource availability, and external pressures—on the relationship between leadership, embeddedness, and innovation is underexplored, highlighting the need for more comprehensive research [33].

## How this study will address the gap

This study aims to fill the gap by empirically examining the impact of nurse managers' boundary-spacing leadership on the relationship between nurses' work embeddedness and innovative work behaviors. By utilizing validated scales and conducting correlation and regression analyses, the study seeks to quantify the influence of boundary-spacing leadership on work embeddedness and innovation. The findings will provide insights into the relative importance of work embeddedness in fostering innovation and clarify the mechanisms through which leadership styles can enhance or hinder this process. This research will contribute to a deeper understanding of how leadership can be leveraged to create a supportive and innovative work environment in healthcare, ultimately leading to better patient outcomes and enhanced nurse retention.

## Significant of the study

Because of increased working hours and lack of organizational system lead to a negative work environment, such as the poor coworkers’ relationships, leading to increased work pressure on nursing and affected the efficiency of work embeddedness and innovative work performance as a team. So, boundary spacing is essential for head nurses since nursing team does not work alone. when nurses have high-quality professional supervision and boundary spacing activities through head nurses' role, they are typically successful and work autonomously [34]. Nursing leadership characteristics are more vital to assist nurses in becoming more innovative; therefore, encouraging innovative work performance among nurses has been an important development direction for boundary spacing leaders [35]. So, this study aims to assess head nurse’s boundary spacing leadership and its effect on nurses’ work embeddedness and innovative work performance.

## Research hypotheses

### H1

A positive relationship exists between nurse managers' boundary spacing leadership and nurses' work embeddedness. (This hypothesis suggests that nurses who perceive their managers as exhibiting strong boundary spacing leadership will feel more attached and connected to their organization).

### H2

A positive relationship exists between nurses' work embeddedness and innovative work behaviors. (This hypothesis suggests that nurses who feel more embedded in their organization will be more likely to engage in innovative work behaviors).

### H3

Nurse managers' boundary-spacing leadership positively affects nurses' innovative work behaviors through work embeddedness as a mediating variable. (This hypothesis suggests that the positive effect of boundary spacing leadership on innovative work behaviors is partially explained by its influence on work embeddedness. In simpler terms, nurse managers who exhibit strong boundary spacing leadership create an environment that fosters work embeddedness, which in turn leads to increased innovative work behaviors among nurses).

### Methods

#### Design

Descriptive correlation was used in the study design. The choice of a descriptive correlational design for this study is justified by its effectiveness in examining the relationships between variables, particularly when the aim is to identify associations rather than establish causality. This design allows researchers to observe and measure the extent to which variables, such as boundary-spacing leadership, work embeddedness, and innovative work behaviors, are related within a natural setting without manipulating the environment. Descriptive correlational studies are particularly suited for exploring the interconnectedness of variables, providing a foundation for understanding how one variable may influence another.

Similar studies in healthcare and organizational behavior have successfully employed correlational designs to explore the relationships between leadership styles and various outcomes, such as job satisfaction, employee retention, and innovative behaviors. For instance, a study by Zhang et al. (2018) utilized a correlational design to examine the relationship between transformational leadership and innovative work behavior among nurses, finding significant positive associations that informed leadership development programs. The theoretical framework underlying the Job Embeddedness Theory [16, 17] also supports the use of correlational designs to explore how embeddedness within an organization influences employee behaviors and attitudes, particularly in complex environments like healthcare.

The descriptive correlational design is thus appropriate for this study, as it facilitates the exploration of how boundary-spacing leadership impacts work embeddedness and, subsequently, innovative work behaviors among nurses. This approach allows for the identification of significant relationships that can inform leadership practices and organizational policies aimed at fostering a culture of innovation in healthcare settings.

## Setting

The study was conducted in all units of a governmental university hospital in Tanta, Egypt. Tanta University Hospital, a prominent governmental institution in Tanta, Egypt, serves as a crucial healthcare provider for the citizens of Tanta and neighboring governorates. The hospital is structured into various specialized units, including critical care, surgical, internal medicine, pediatric, and obstetrics and gynecology units, each equipped with advanced medical technologies and staffed by highly trained professionals. Additionally, the hospital operates numerous outpatient clinics, which serve as primary points of contact for patient consultations, diagnostics, and minor procedures. Beyond its clinical services, Tanta University Hospital plays a vital educational role, offering medical and nursing students extensive opportunities for practical experience under the supervision of seasoned healthcare professionals. The hospital is also a key site for research, hosting numerous projects aimed at advancing medical science and improving healthcare practices. The study conducted across all units of Tanta University Hospital in November and December 2023 adhered to the Strengthening the Reporting of Observational Studies in Epidemiology (STROBE) guidelines, ensuring the rigor and quality of the research. This combination of comprehensive clinical services, educational opportunities, and research activities underscores the hospital’s significant contribution to both patient care and the professional development of healthcare practitioners in the region.

### Sample size calculation

The sample size calculation was performed using Epi-info 7 software, a widely accepted tool for epidemiological research. The parameters used were carefully chosen to ensure statistical validity: The anticipated prevalence or occurrence of the phenomenon under investigation was 50%. This was chosen as a conservative estimate to ensure that the sample size would be adequate regardless of the true frequency. The choice of a 50% prevalence estimate in the sample size calculation for the study was strategically adopted as a conservative measure to ensure the robustness and validity of the research findings. By selecting a 50% prevalence, the researchers ensured that the sample size would be sufficient to detect significant effects under the most variable conditions, thereby maximizing statistical power and accuracy. The use of Epi-info 7 software for sample size calculation, coupled with a 95% confidence level and a permissible margin of error of 5%, underscores the commitment to achieving precise and reliable results. The final sample size of 397, adjusted for a 10% dropout rate, was calculated to accommodate an effect size of 0.03 and a power of 80%, ensuring the study's ability to detect meaningful differences. This conservative approach mitigates the risk of underestimating the required sample size, thereby enhancing the generalizability and practical implications of the study's outcomes. The level of confidence desired in the study results is 95%. This indicates a 95% probability that the true population parameter lies within the calculated confidence interval. The maximum permissible difference between the sample estimate and the true population parameters is 5%. This ensures that the sample estimate remains sufficiently close to the true value for practical purposes. The study included nurses who agreed to participate, had at least a year of clinical experience, and worked full-time in the aforementioned setting. The total population was 1400 nurses, and the sample size of 380 nurses with an effect size of 0.03, a type 1 error of 0.05, a power of 80%, and three predictors was determined using G*power (version 3.1.9.7). Considering a 10% dropout rate, the sample size was increased to 397. A total of 373 nurses were assessed for eligibility, and a convenience sample of 250 was ultimately analyzed.

### Inclusion criteria

- Nurses with more than one year experience in the study setting.
- Nurses who voluntarily agree to take part in the research study after being provided with information about its purpose, procedures, potential risks, and benefits.

### Exclusion criteria

- Newly hired nurse with less than 1 year of nursing experience.
- Nurses who do not provide their voluntary assent to participate in the study.

### Study instruments

A questionnaire was used in this study. There were two sections to the survey. Sociodemographic and work-related characteristics, including age, gender, marital status, educational qualifications, working unit, years of experience as a nurse, years of experience at the unit.

The second section included three self-reported questionnaires were used to collect data about the study variables.

### Tool I: nurse managers boundary spacing scale (NMBS)

Nurses' perception of their nurse managers’ boundary spacing leadership was measured using a 26 items scale created by Onishi et al., [36],  Onishi [36]. The scale consisted of three dimensions; connecting and mediating (13 items), informing and feedback utilization (8 items), and resource acquisition (5 items). A 5-point Likert scale (1 being strongly disagree and 5 being strongly agree) was used to rate the scale’s items. A higher scale score indicates a higher level of nurses' perception of their nurse managers’ use of boundary-spacing leadership. The reliability coefficient of the scale was 0.893, indicating the scale's high internal consistency.

### Tool II: The global job embeddedness scale (JES)

The seven-item job embeddedness scale was used to assess nurses’ level of embeddedness in their work [37]). On a Likert scale that ranges from 1 (strongly disagree) to 5 (strongly agree), each respondent scored each of the six statements, and item 6 was scored in reverse. Lower scores suggested a lesser level of nurses’ work embeddedness, while higher scores indicated potentially higher level of nurses’ work embeddedness. The reliability coefficient of the scale was 0.863, indicating the scale's acceptable internal consistency.

### Tool III: innovative work behavior scale (IWBS)

Nurses’ perception of their innovative work behavior was measured with the ten-item scale reported in the work of De Jong and Den Hartog [38], De Jong et al. [38]. Likert scale that ranges from 1 (never) to 5 (always), with a high score indicating higher level of innovative work behavior. The scale yielded adequate internal reliability, with α = 0.945.

### Validity and reliability

In order to accommodate Egyptian culture, the study materials were translated from English into Arabic following the back translation process [39]). This was carried out to ensure accuracy and any possible risks to the validity of the study. After the instruments were translated, their validity was examined by a panel of five professors of nursing administration. The word-choices identified by the expert panel were changed in accordance with their suggestions. A pilot study with 25 nurses was conducted to verify the instruments' accuracy and usability and determine the amount of time needed to complete the questionnaire. This pilot sample was not included in the final analysis.

Cronbach's alpha was used to assess the instruments' internal consistency. The scales measuring boundary spacing leadership, work embeddedness, and innovative work behavior Cronbach's alphas of .893, .863, and .945, respectively. Accordingly, these findings show that the three study tools' internal consistency was satisfactory.

### Data collection

The researchers collected the data using a hand-delivered anonymous questionnaire to eligible nurses. The average time to complete the questionnaire was twenty minutes. Data was collected for two months, from November 1st, 2023, to December 30th, 2023. Every participant's query was fully addressed and made clear.

### Ethical consideration

The medical and nursing administrations of the study setting gave their written approval for the study. The study was approved by Tanta University- Faculty of Nursing Scientific Research Ethical Committee (IRB:317-10-2023–29/10/2023) in order to collect the required data. The participating nurses received a detailed explanation of the study's objectives, and assurances that the data would only be used for research. They were aware that there would be no consequences if they chose not to participate in the study or left before submitting the necessary paperwork. Nurses who volunteered to participate in the study gave informed consent. The researchers respected and considered everyone's right to privacy. During the study, data privacy was protected as one of the researchers coded and protected all responses. Potential biases in the current study were mitigated by the researchers as we recruited participants from diverse units within the hospital and applied stringent eligibility criteria to ensure relevant experience. We also maintained transparency by documenting the sampling process and demographic details.

### Statistical analysis

Analyses in this study were performed using IBM SPSS version 28 and AMOS version 25. Following data collection, the data were coded, modified, and imported into these statistical applications. Reliability of the instruments was assessed using Cronbach's alpha. Descriptive statistics were employed to summarize the data: frequency tables and cross-tabulations illustrated categorical data, while numerical data were described using the mean, standard deviation, minimum, and maximum values. For categorical variables, percentages were used to characterize the frequency distribution of each category.

Inferential statistical analyses were conducted to explore the relationships between variables. The Pearson correlation test assessed the strength and direction of the relationships among boundary spanning leadership, work embeddedness, and innovative work behavior. Correlation coefficients were interpreted as weak (near 0), moderate (around 0.50), or strong (near − 1 or 1), with *p*-values ≤ 0.05 indicating statistical significance. Additionally, linear regression analysis was used to model the relationship between two variables by fitting a linear equation to the data. The inter-correlation among research variables was examined using Pearson pairwise correlations, with significance set at the 5% level (*p*-value < 0.05). SEM was applied to test the measurement model and investigate the mediating effects of variables, with goodness-of-fit assessed using criteria such as χ2/df < 3, RMSEA < 0.08, and TLI, IFI, and CFI ≥ 0.90.

## Results

The data presented in Table 1 provides a demographic overview of the nurses included in the study. The largest age group among the nurses was those between 20 and 29 years old, making up 38.4% of the sample, suggesting a relatively young nursing workforce. An overwhelming majority of the nurses were female, comprising 96.0% of the sample, reflecting the gender imbalance commonly observed in the nursing profession. A significant portion of the nurses were married, with 89.2% reporting this status, indicating that most nurses in the study have family responsibilities. Regarding, educational background, 67.6% of the nurses held a bachelor’s degree in nursing, indicating a high level of formal education within the group, while a small fraction, 1.2%, had attained a PhD, suggesting that advanced academic qualifications are relatively rare among these nurses. Regarding professional experience, about one third of the nurses (34.0%) had been in the field for 11 to 20 years, indicating a substantial proportion of the nurses have significant work experience, potentially contributing to their professional competence and stability.

**Table 1:** Sociodemographic characteristics of the study participants (*n* = 250)

Demographic data	No	%
**Age**		
20–29	96	38.4
30–39	89	35.6
40–49	60	24.0
≥ 50	5	2.0
Mean ± SD	33.83 ± 7.51	
**Gender**		
Male	10	4.0
Female	240	96.0
**Marital status**		
Single	20	8.0
Married	223	89.2
Divorced	5	2.0
Widowed	2	.8
**Educational Qualification**		
Secondary nursing school	7	2.8
Nursing institute	52	20.8
Bachelor	169	67.6
Master	19	7.6
PhD	3	1.2
**Working Unit**		
Anesthesia	1	0.4
CCU	5	2.0
Economy	4	1.6
Education	4	1.6
Emergency	15	6.0
Sterilization	7	2.8
Head nurse	2	0.8
Hemodialysis	8	3.2
ICU	41	16.4
Internal	19	7.6
NICU	7	2.8
Nursing admiration	25	10.0
Outpatients	3	1.2
PICU	8	3.2
Quality	10	4.0
Surgery	39	15.6
Ward	52	20.8
**Years of experience as a nurse**		
< 5	66	26.4
5–10	58	23.2
11–20	85	34.0
> 20	41	16.4
**Mean ± SD**	11.73 ± 7.02	
**Years of experience in the unit**		
< 5	153	61.2
5–10	60	24.0
11–20	27	10.8
> 20	10	4.0
**Mean ± SD**	10.19 ± 6.55	
Demographic data	No	%
**Mean ± SD**	10.19 ± 6.55	

The results presented in Table 2 provide insights into nurses' perceptions of work embeddedness. The overall mean score for nurses' perception of work embeddedness was 3.56, with a standard deviation of 0.77. This indicates that, on average, nurses have a moderately positive perception of their embeddedness in their work environment, though there is some variability in their responses. Less than half of the nurses (48.4%) had a moderate perception of work embeddedness, while about one third (32.4%) expressed a high perception of work embeddedness. This suggests that while a significant portion of nurses feel moderately connected to their organization, a substantial number feel highly embedded. The highest mean score (4.00 ± 1.00) was associated with the item "I feel attached to this organization," indicating that a strong attachment to the organization is a key aspect of work embeddedness for many nurses. Over two-thirds of the study sample (75%) reported a high perception of this aspect of work embeddedness. Conversely, the item "I’m too caught up in this organization to leave" had the lowest mean score (3.44 ± 1.02), with more than one third (43.6%) of the nurses expressing a high perception. This lower score suggests that feeling entrapped by the organization is less prevalent among nurses compared to feelings of attachment. Overall, these findings highlight that while nurses have a moderate to high level of work embeddedness, the sense of attachment to the organization is particularly strong, whereas feelings of being too entangled to leave are less pronounced.

**Table 2:** Descriptive analysis of studied nurses' perception of their Work Embeddedness (*n* = 250)

	Nurses’ Work Embeddedness	Mean ± SD			Low		Moderate		High	
					No.	%	No.	%	No.	%
1	I feel attached to this organization.	4.00	±	1.00	22	8.8	40	16.0	188	75.2
2	It would be difficult for me to leave this organization.	3.67	±	1.11	44	17.6	61	24.4	145	58.0
3	I’m too caught up in this organization to leave.	3.44	±	1.02	45	18.0	96	38.4	109	43.6
4	I feel tied to this organization.	3.79	±	0.96	27	10.8	55	22.0	168	67.2
5	I simply could not leave the organization that I work for.	3.62	±	1.07	46	18.4	67	26.8	137	54.8
6	It would be easy for me to leave this organization (reversed item)	2.73	±	1.03	114	45.6	89	35.6	47	18.8
7	I am tightly connected to this organization.	3.65	±	1.06	39	15.6	61	24.4	150	60.0
	**Overall Nurses’ Work Embeddedness**	**3.56**	**±**	**0.77**	**48**	**19.2**	**121**	**48.4**	**81**	**32.4**

The data in Table 3 sheds light on nurses' perceptions of their managers' boundary-spacing leadership. The average score (3.78) suggests a moderate level of effectiveness, with some variation in responses. While nearly half the nurses (44.4%) perceive this leadership as moderate, another significant portion (40.8%) view it highly. Digging deeper into specific areas, we see a range of perceptions. Connecting and mediating across the organization seems less prevalent, with only 25.8% of nurses perceiving high effectiveness. In contrast, resource acquisition appears to be a strength, with nearly half (48.8%) of nurses seeing their managers as highly proficient. Information and feedback management also fares well, with 46.4% of nurses perceiving high effectiveness. These findings point to a generally positive perception of nurse managers' boundary-spacing leadership, particularly in resource acquisition and information sharing. However, there's opportunities for improvement in connecting and mediating within the organization.

**Table 3:** Descriptive analysis of studied nurses' perception of nurse managers’ boundary spacing leadership (*n* = 250)

Head Nurses Boundary Spacing leadership	Mean ± SD			Low		Moderate		High	
				No.	%	No.	%	No.	%
Connecting and mediating	3.85	±	0.92	32	12.8	86	34.4	132	52.8
Informing and feedback utilization	3.78	±	0.84	32	12.8	102	40.8	116	46.4
Resource acquisition	3.71	±	0.93	30	12.0	98	39.2	122	48.8
**Overall Head Nurses Boundary Spacing leadership**	**3.78**	**±**	**0.85**	**37**	**14.8**	**111**	**44.4**	**102**	**40.8**

The results presented in Table 4 indicate that a majority of nurses (52.0%) had a high perception of their innovative work performance, demonstrating a strong overall engagement in innovation within their roles. In contrast, only a small percentage (7.2%) expressed a low perception of their innovative capabilities. When examining specific subscales, the data reveals that 68.0% of nurses had a high perception of idea generation, 67.2% had a high perception of idea implementation, 64.0% had a high perception of idea championing, and 47.6% had a high perception of idea exploration. These findings suggest that nurses feel particularly confident and engaged in generating, implementing, and championing new ideas, although there is slightly less confidence in exploring new ideas. Overall, the data highlights a positive trend in nurses' perceptions of their innovative work behaviors, with strong engagement in various stages of the innovation process.

**Table 4:** Descriptive analysis of studied nurses' perception of Innovative Work Performance (*n* = 250)

Innovative Work Performance	Mean ± SD			Low		Moderate		High	
				No.	%	No.	%	No.	%
Idea exploration	3.70	±	0.76	23	9.2	108	43.2	119	47.6
Idea generation:	4.05	±	0.76	12	4.8	68	27.2	170	68.0
Idea championing	3.90	±	0.87	19	7.6	71	28.4	160	64.0
Idea Implementation:	3.99	±	0.87	17	6.8	65	26.0	168	67.2
**Overall Innovative Work Performance**	**3.91**	**±**	**0.74**	**18**	**7.2**	**102**	**40.8**	**130**	**52.0**

The findings in table 5 reveal a significant positive relationship between nurses' innovative work performance and their work embeddedness, with a correlation coefficient (r) of 0.267, *p* < 0.001. This indicates that as nurses feel more embedded in their work environment, their innovative performance tends to increase. Additionally, a stronger positive relationship exists between innovative work performance and nurse managers’ boundary-spacing leadership, with a correlation coefficient (r) of 0.421, *p* < 0.001. Furthermore, there is a significant positive correlation between nurse managers’ boundary-spacing leadership and nurses work embeddedness with a correlation coefficient (r) of 0.615, *p* < 0.001. This suggests that effective boundary-spacing leadership by nurse managers is associated with higher levels of innovative work performance among nurses. These results underscore the importance of both work embeddedness and supportive leadership in fostering innovation within the nursing workforce.

**Table 5:** Correlation table between the study variables

		Nurses’ Work Embeddedness	Connecting and mediating	Informing and feedback utilization	Resource acquisition	Overall Nurse Managers Boundary Spacing leadership	Idea exploration	Idea generation	Idea championing	Idea Implementation	Overall Innovative Work Performance
**Nurses’ Work Embeddedness**	r										
	p										
**Connecting and mediating**	r	0.622^*^									
	p	< 0.001^*^									
**Informing and feedback utilization**	r	0.554^*^	0.869^*^								
	p	< 0.001^*^	< 0.001^*^								
**Resource acquisition**	r	0.565^*^	0.800^*^	0.857^*^							
	p	< 0.001^*^	< 0.001^*^	< 0.001^*^							
**Overall Nurse Managers Boundary Spacing leadership**	r	0.615^*^	0.942^*^	0.958^*^	0.938^*^						
	p	< 0.001^*^	< 0.001^*^	< 0.001^*^	< 0.001^*^						
**Idea exploration**	r	0.152^*^	0.221^*^	0.315^*^	0.352^*^	0.313^*^					
	p	0.016^*^	< 0.001^*^	< 0.001^*^	< 0.001^*^	< 0.001^*^					
**Idea generation**	r	0.241^*^	0.313^*^	0.389^*^	0.406^*^	0.390^*^	0.675^*^				
	p	< 0.001^*^	< 0.001^*^	< 0.001^*^	< 0.001^*^	< 0.001^*^	< 0.001^*^				
**Idea championing**	r	0.285^*^	0.301^*^	0.418^*^	0.445^*^	0.410^*^	0.676^*^	0.866^*^			
	p	< 0.001^*^	< 0.001^*^	< 0.001^*^	< 0.001^*^	< 0.001^*^	< 0.001^*^	< 0.001^*^			
**Idea Implementation**	r	0.277^*^	0.301^*^	0.407^*^	0.447^*^	0.406^*^	0.651^*^	0.839^*^	0.833^*^		
	p	< 0.001^*^	< 0.001^*^	< 0.001^*^	< 0.001^*^	< 0.001^*^	< 0.001^*^	< 0.001^*^	< 0.001^*^		
**Overall Innovative Work Performance**	r	0.267^*^	0.315^*^	0.424^*^	0.458^*^	0.421^*^	0.822^*^	0.933^*^	0.937^*^	0.924^*^	
	p	< 0.001^*^	< 0.001^*^	< 0.001^*^	< 0.001^*^	< 0.001^*^	< 0.001^*^	< 0.001^*^	< 0.001^*^	< 0.001^*^	

*r* Pearson coefficient

*Statistically significant at *p* ≤ 0.05

**Fig. 2:**
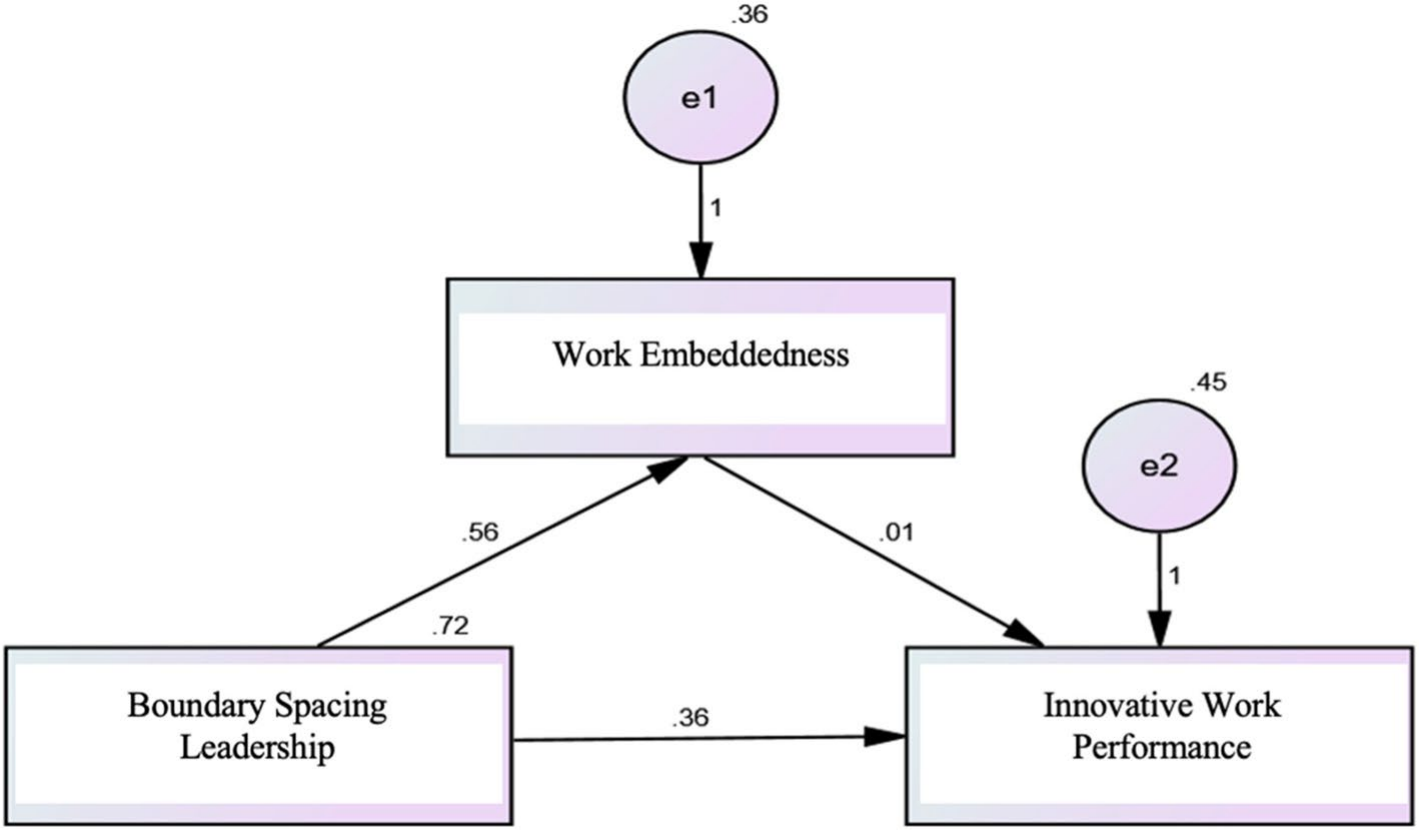
Structure Equation Modeling **(n = 250). **Model fit parameters CFI; IFI; RMSEA (1.000; 1.000; 0.409). CFI = Comparative fit index; IFI = incremental fit index; and RMSEA = Root Mean Square Error of Approximation. Model c^2^; significance 55.643^*^(<0.001^*^)

The results depicted in Fig. 2, using structural equation modeling (SEM), demonstrate the standardized regression weights along with model fit parameters. The model fit indices indicate a good fit, with a Comparative Fit Index (CFI) and Incremental Fit Index (IFI) both equal to 1.000, suggesting that the model accurately represents the data. However, the Root Mean Square Error of Approximation (RMSEA) is 0.409, which is higher than the commonly accepted threshold, indicating some degree of model misfit. The Chi-square (X²) significance is 55.643 with a *p*-value of less than 0.001, further supporting the model's statistical significance. Boundary-spacing leadership explained 56% of the variance in work embeddedness and 36% in nurses' innovative work performance. These results underscore the critical role of boundary-spacing leadership in enhancing both the work embeddedness and the innovative behaviors of nurses. The significant portion of variance explained in these outcomes highlights the importance of effective leadership practices in promoting a supportive and innovative work environment within the nursing field.

The results in Tables 6 and 7 demonstrate significant positive direct effects of nurse managers' boundary-spacing leadership on both work embeddedness and innovative work performance among nurses. In Table 6, the direct effect of boundary-spacing leadership on work embeddedness is 0.556, with a *p*-value of 0.001, indicating that effective boundary-spacing leadership significantly contributes to nurses feeling more embedded in their work environment. Similarly, in Table 10, the direct effect of boundary-spacing leadership on innovative work performance is 0.362, with a *p*-value of less than 0.001, highlighting the crucial role of leadership in fostering innovative behaviors among nurses. Additionally, there is an indirect effect of nurse managers' boundary-spacing leadership on innovative work performance, with a coefficient of 0.06. This indirect effect suggests that boundary-spacing leadership influences innovative work performance through pathways that are not directly measured in the model, potentially involving factors such as improved communication, resource access, or team collaboration facilitated by the manager's boundary-spacing activities. Overall, these findings underscore the importance of boundary-spacing leadership in enhancing both work embeddedness and innovative work performance among nurses, emphasizing the multifaceted impact that effective leadership can have on organizational outcomes in healthcare settings.

**Table 6:** Direct and Indirect Effect

Variable 1		Variable 2	Standardized regression weights	S.E	C.R	p-value
*Work Embeddedness*	←	**Nurse Managers Boundary Spacing leadership**	0.556	0.045	12.302^*^	0.001^*^
*Innovative Work Performance*	←	**Nurse Managers Boundary Spacing leadership**	0.362	0.064	5.681^*^	< 0.001^*^
*Innovative Work Performance*	←	**Work Embeddedness**	0.012	0.070	0.165	0.869

**Table 7:** Direct and Indirect effect

Variables		Direct effect	Indirect effect	CI			p-value
*Work Embeddedness* - >	**Nurse Managers Boundary Spacing leadership**	0.556	0.0	0.544	-	0.568	0.001^*^
*Innovative Work Performance* - >	**Nurse Managers Boundary Spacing leadership**	0.362	0.06	0.059	-	0.783	< 0.001^*^
*Innovative Work Performance* - >	**Work Embeddedness**	0.012	0.0	-0.603	-	0.627	0.869

## Discussion

This research explored the impact of nurse managers' boundary-spacing leadership on the relationship between nurses' work embeddedness and their innovative work behaviors within Egyptian hospitals. Boundary-spacing leadership involves bridging organizational boundaries to enhance staff performance and foster innovation. The study aimed to determine how this leadership style influences the connection between nurses’ sense of workplace connection (work embeddedness) and their innovative behaviors.

The findings reveal that while all three variables—boundary-spacing leadership, work embeddedness, and innovative work behaviors—were positively correlated, the influence of boundary-spacing leadership on innovative work behaviors was particularly strong. This suggests that effective boundary-spacing leadership directly promotes innovative behaviors among nurses. However, boundary-spacing leadership had a weak and non-significant effect on work embeddedness, indicating that enhancing leadership skills in this area may not necessarily improve nurses' sense of connection to their workplace.

This finding is consistent with research by De Regge et al. [2], which highlights that boundary-spacing behaviors positively influence nurses' commitment to their organization but does not always directly enhance work embeddedness. Moreover, [40] emphasized that while managerial support for boundary-spacing behaviors is crucial for innovation, the direct impact of such leadership on work embeddedness can be limited.

Additionally, the study found that work embeddedness had a minimal direct effect on innovative behaviors. This suggests that feeling connected to the workplace does not directly drive innovation in this context. Previous studies support this notion, indicating that innovative work behaviors are more strongly influenced by leadership and organizational support rather than by work embeddedness alone [5, 6].

Future research should investigate the underlying mechanisms that explain these relationships. Specifically, it would be valuable to explore whether aspects of boundary-spacing leadership, such as frequent role transitions or limited interaction with core team members, could impact nurses' sense of connection to their workplace. Research by Xie et al. [41] supports the idea that low managerial self-efficacy and ineffective boundary-spacing behaviors can hinder organizational cohesion and innovation. Understanding these dynamics can offer deeper insights into how boundary-spacing roles influence work embeddedness and contribute to organizational culture.

The specific work environment of the participants, primarily in intensive care units (ICUs) and operating theaters, may have influenced the study’s outcomes. These settings inherently support effective boundary-spacing leadership due to the need for strong collaboration, information exchange, and resource acquisition [42]. These environments may also require nurses to participate in decision-making processes, further enhancing the impact of boundary-spacing leadership on innovative work behaviors.

This study builds on existing literature by examining the effectiveness of boundary-spacing leadership in nursing. Previous research highlights the role of healthcare organizations, supervisors, and colleagues in fostering boundary-spacing behaviors [2]. Additionally, [43] introduced bounded leadership theory, emphasizing that leader competencies alone are insufficient without considering organizational constraints.

Furthermore, the interplay between boundary-spacing strategies and their effects on boundary work has been explored by Fick-Cooper [6] and Kislov [5], highlighting the complex dynamics of boundary work. Xie et al. [41] also emphasize that low managerial self-efficacy can negatively impact boundary-spacing behaviors, suggesting the need for supportive work contexts to encourage such behaviors.

Despite the moderate ratings of work embeddedness and innovative work behaviors among nurses, these perceptions may be influenced by factors such as age, education, department, and experience. Nurses' moderate involvement in external activities and decision-making processes within the hospital could also affect their sense of work embeddedness and innovation. This aligns with findings by Karatepe and Neche [13], which suggest that moderate work embeddedness is common among nurses.

Finally, the study by [42] demonstrates the relevance of boundary-spacing leadership during the COVID-19 pandemic, where such leadership facilitated innovation in response to the crisis. This underscores the critical role of boundary-spacing leadership in navigating and driving advancements in healthcare.

In summary, while boundary-spacing leadership is crucial for promoting innovative work behaviors, its impact on work embeddedness is limited. Future research should explore how boundary-spacing leadership influences work embeddedness and investigate additional factors that contribute to a culture of innovation in nursing teams.

### Limitations

It is important to note the limitations of this study. The cross-sectional and single-source data may be biased. Data from various sources (such as supervisor ratings of the attitudes and behaviors of the focal respondents) may be gathered in future investigations. Additionally, the authors acknowledge the cross-sectional design's limitations regarding assessing causal links between the variables. A longitudinal research design is necessary to support this idea. Utilizing an experimental design is a further suggestion. Future research may concentrate on boundary spacing leadership, work embeddedness and innovative work behavior in various national and cultural contexts. Finally, our research is limited in the studied sample, restricting the generalizability of the findings. As is known for us, as Egyptians with a different culture, and thus it may not fit in western context. To tackle this problem, cross-culture investigation would be useful in pinpointing the applicability of our study results.

## Conclusion

This study investigated the factors influencing nurses' innovative work performance. The findings highlight nurse managers' significant role in boundary-spacing leadership and nurses' work embeddedness. The study identified nurse managers' boundary spacing leadership as the strongest predictor of nurses' innovative work performance. This leadership style involves connecting internal and external resources, fostering information exchange, and advocating for the nursing team. While work embeddedness positively correlated with innovative work performance, its impact was less significant than boundary-spacing leadership. This suggests that feeling attached to the organization fosters innovation, but leadership style has a stronger influence. Furthermore, the study found a positive and significant correlation between innovative work performance and both work embeddedness and nurse managers' boundary spacing leadership.

### Implications in nursing practice

This study offers valuable insights for improving healthcare innovation through leadership and staff engagement. The findings highlight the importance of nurse managers developing their "boundary spacing" leadership skills. This translates to fostering collaboration within the team, actively advocating for resources to support nurses' work, and ensuring effective communication and information flow. These actions create an environment that encourages and empowers nurses to innovate. Additionally, strategies that enhance nurses' sense of connection to the organization, such as recognition programs and opportunities for professional development, can further contribute to a culture of innovation within the healthcare setting. Building upon the foundation of boundary spacing leadership and work embeddedness, nurse managers can further cultivate a thriving culture of innovation through a multi-pronged approach. This includes creating safe brainstorming and open communication spaces, where "out-of-the-box" thinking is celebrated. Additionally, allocating resources and time for nurses to pilot their innovative ideas on a small scale allows for testing and refinement, reducing the fear of failure associated with large-scale changes. Recognizing and rewarding innovative work publicly motivates others and reinforces its value. Furthermore, nurse managers can be crucial in connecting nurses with external resources such as professional organizations, research opportunities, or collaborations with other healthcare institutions. Finally, mentorship and coaching opportunities empower nurses to develop their creative problem-solving skills and refine their innovative ideas. Future research should consider the potential effects of nurse managers’ boundary-spacing leadership on workplace violence and nurse’s quality of life [44, 45]. By implementing these strategies, nurse managers can create a powerful environment that fosters and sustains a culture of innovation within their teams, ultimately leading to improved patient care and better health outcomes.

## Data Availability

Data will be available on reasonable request from the corresponding author.
